# Relationships among anxiety, psychological resilience, and physical activity in university students: variable-centred and person-centred perspectives

**DOI:** 10.3389/fpsyg.2025.1694344

**Published:** 2025-11-21

**Authors:** Yuqing Yuan, Jing Yang, Wenying Huang, Chang Hu, Wen Zhang, Bin Chen

**Affiliations:** 1 Physical Education College, Jiangxi Normal University, Nanchang, China; 2 Mianyang Normal University, Mianyang, China

**Keywords:** anxiety, psychological resilience, physical activity, university students, mediation analysis, latent profile analysis

## Abstract

**Background:**

Anxiety is significantly correlated with levels of physical activity in university students. This research assessed the effects of anxiety on engagement in physical activity and explored the potential mediating function of psychological resilience. Additionally, latent profile analysis (LPA) was employed to identify distinct subtypes based on anxiety and resilience levels, and to explore their associations with physical activity.

**Methods:**

Utilizing a non-probability convenience sampling approach, this cross-sectional study recruited a total of 1,436 collegiate participants from multiple universities. Data collection was carried out with the Generalized Anxiety Disorder Scale (GAD-7), the abbreviated Connor-Davidson Resilience Scale (CD-RISC-10), and the Physical Activity Rating Scale (PARS-3). Data analysis included mediation effect analysis via Bootstrap methods (Model 4) and latent profile analysis (LPA).

**Results:**

Anxiety demonstrated a significant negative association with physical activity (*β* = −0.307, *p* < 0.001) and was identified as a substantial negative predictor of psychological resilience (*β* = −0.413, *p* < 0.001). A significant positive association was observed between psychological resilience and physical activity (*β* = 0.178, *p* < 0.001). The analysis confirmed the role of psychological resilience as a significant mediating variable in the pathway linking anxiety to physical activity, accounting for 24% of the total effect. Furthermore, latent profile analysis identified three distinct typologies: High Anxiety-Low Psychological Resilience (HA-LPR; 10.38%), Moderate Anxiety-Moderate Psychological Resilience (MA-MPR; 62.74%), and Low Anxiety-High Psychological Resilience (LA-HPR; 26.88%). These latent classes demonstrated statistically significant differences in physical activity levels (*F* = 209.655, *p* < 0.001).

**Conclusion:**

Results demonstrated that anxiety affects physical activity both directly and indirectly, with the latter effect occurring through the channel of psychological resilience. Latent profile analysis identified three distinct profiles among college students based on anxiety and psychological resilience: High Anxiety-Low Psychological Resilience, Moderate Anxiety-Moderate Psychological Resilience, and Low Anxiety-High Psychological Resilience. Marked variations in physical activity levels were observed among these subgroups. The results underscore the complex relationships among mental health indicators and health behaviors within the collegiate population. The delineation of distinct profiles offers practical implications for designing tailored intervention strategies.

## 1 Introduction

Mental health is widely acknowledged as a fundamental component of individual well-being (Campbell et al., 2022; Yang et al., 2025). Anxiety has been recognized by leading authorities, such as the World Health Organization (WHO), as one of the most pressing public health challenges worldwide (Li J. et al., 2024; Liu and Wang, 2024). University students are considered a particularly vulnerable demographic (Harith et al., 2022; Jones et al., 2018; DiFonte et al., 2024; Piña-Watson et al., 2025). Empirical longitudinal data demonstrate a marked elevation in the incidence of clinical anxiety among university students when compared to non-academic populations of comparable age (McCloud et al., 2023). Recent network analyses focusing on Chinese student populations further indicate that anxiety may have exceeded depression as the central feature within networks of internalizing psychopathology (Li J. et al., 2024), with estimated prevalence rates reaching 39.0% (Li et al., 2022). Concurrently, insufficient physical activity (PA) is a pervasive public health issue within the university student demographic (Fagaras et al., 2015; Hao et al., 2022; Li B. et al., 2024; Strain et al., 2024), and anxiety has emerged as a key psychological predictor hindering regular PA engagement in this group (Mercader-Rubio et al., 2023; Li X. et al., 2024; Beenen et al., 2025). Consequently, elucidating the mechanistic pathways through which anxiety impedes physical activity in this population constitutes a pressing public health priority.

The extant literature substantiates a consistent inhibitory effect of anxiety on PA participation (Babaev et al., 2018; Beltrán et al., 2024); however, the underlying mechanisms, particularly potential mediating pathways, remain inadequately investigated. Psychological resilience represents a promising protective factor that may mitigate the adverse effects of anxiety on PA participation (Lau, 2022; Xu et al., 2023). Furthermore, the predominant reliance on variable-centered methodologies has characterized prior investigations into the connections between anxiety, psychological resilience, and PA (Liu et al., 2021; Liao et al., 2023). A common limitation of these methodologies is their tendency to overlook the heterogeneous and idiosyncratic nature of how anxiety and psychological resilience manifest across individuals (Newland et al., 2013; Sun et al., 2023; Yöyen et al., 2024). Moreover, extant studies typically treat anxiety and psychological resilience as continuous composite scores (Hou et al., 2021), thereby masking the possibility of aspects of these influences on PA engagement. In response to these limitations, a combined variable-centered and person-centered analytical approach is employed in the current investigation. The primary objective is to elucidate the relationship between anxiety and PA within a collegiate population, placing specific emphasis on the mediating effects attributable to psychological resilience. Using Latent Profile Analysis (LPA) (He and Fan, 2019), we identify homogeneous subgroups based on individual patterns of anxiety and psychological resilience, and subsequently examine how these distinct profiles are associated with varying levels of PA engagement.

## 2 Theory and hypothesis

### 2.1 Anxiety and physical activity

Anxiety is a prevalent emotional disorder characterized by persistent, excessive, and difficult-to-control feelings of worry, fear, or tension in response to perceived threats or stressful situations (Farchione et al., 2012; Stults-Kolehmainen and Sinha, 2014). Somatic symptoms frequently emerge before emotional awareness in these manifestations (Penninx et al., 2021). Physical activity encompasses all voluntary bodily movements driven by skeletal muscles, which necessitate energy expenditure above the body’s baseline level (Bull et al., 2020). Research has well-established an inverse relationship between anxiety and physical activity participation (Petruzzello, 2018; Wanjau et al., 2023). Neurophysiological studies demonstrate that anxiety activates the sympathetic nervous system, prompting stress reactions that are often expressed through somatic manifestations, including cardiac palpitations, elevated skeletal muscle tone, and persistent fatigue (Hoehn-Saric and McLeod, 2000; Bigalke and Carter, 2021). These physiological alterations directly increase perceived discomfort during physical exertion, thereby reducing motivation for PA. Furthermore, prolonged anxiety depletes metabolic reserves, resulting in a state of exhaustion that significantly impairs the physiological capacity necessary to maintain regular physical activity (Mariotti, 2015; Vancampfort et al., 2017). This process perpetuates a maladaptive cycle of physical inactivity.

### 2.2 The mediating role of psychological resilience

Conceptualized as a dynamic, developmental process, psychological resilience is characterized by the ability to achieve positive psychological adaptation and preserve functional stability despite exposure to severe traumatic events or prolonged stressors (Fletcher and Sarkar, 2013; Troy et al., 2023). As conceptualized within cognitive-behavioral theory (Kendall, 1993), cognition, emotion, and behavior function as interconnected systems that dynamically influence one another. Psychological resilience, as a function within the cognitive domain, exerts a significant influence on physical activity (Ben-Eliyahu et al., 2018; Yu and Ye, 2023; Wiedenman et al., 2024). A systematic review of 21 independent investigations confirmed a significant reciprocal association between psychological resilience and physical activity, with the collective findings underscoring a reliable positive effect of psychological resilience on PA engagement (Qiu et al., 2025). Research indicates a reliable inverse association between anxiety levels and psychological resilience among collegiate subjects. Specifically indicating that elevated anxiety levels correspond to diminished psychological resilience capacities (Öztekin et al., 2025). This predictive effect remains robust in non-clinical cohorts (Ran et al., 2020). Consequently, it is proposed that resilience constitutes a key mediating pathway through which anxiety symptomatology influences participation in physical activity.

### 2.3 The present study

Collectively, a primary objective of this research is to test whether psychological resilience mediates the relationship between anxiety symptoms and physical activity engagement. Moreover, to identify distinct patterns of association among anxiety, psychological resilience, latent classes, and physical activity engagement in university students using LPA. This combined analytical approach not only contributes to the existing body of literature but also provides novel translational insights aimed at promoting physical activity within collegiate populations. Building upon the established theoretical and empirical evidence, we posit three hypotheses: (H1) Psychological resilience serves as a significant mediator in the relationship linking anxiety to physical activity. (H2) Distinct latent classes emerge from anxiety and resilience configurations in university students. (H3) These anxiety and psychological resilience typologies demonstrate differential effects on physical activity.

## 3 Materials and methods

### 3.1 Participants

This cross-sectional study aims to investigate the relationships between anxiety, psychological resilience, and physical activity among university students. An *a priori* power calculation was performed with G*Power 3.1 for a multiple regression analysis incorporating two predictors. Parameters were set to detect a moderate effect (f^2^ = 0.15) at *α* = 0.05 with 95% power, yielding a required sample size of 107 (Brydges, 2019). To accommodate potential attrition or missing responses, the target recruitment was raised to approximately 120 participants, thereby maintaining statistical power ≥ 0.95. Following prevailing methodological conventions in psychological studies, a minimum sample size equivalent to 10 times the number of survey items was applied (Nicolaou and Masoner, 2013). Given the 20-item instrument utilized herein, a sample of no fewer than 200 respondents was deemed necessary. Integrating these powers and methodological requirements, a final minimum sample of 200 was established.

Participant recruitment was conducted between March and June 2025 using convenience sampling on the www.wjx.cn platform. Upon securing institutional approvals, course instructors at four Jiangxi Province universities were briefed and asked to share an anonymized QR code with their class WeChat groups and personal social networks, facilitating direct access to the electronic questionnaire (Jiangxi Normal University, Nanchang Normal University, East China Jiaotong University, Jiangxi Science and Technology Normal University). Eligibility for study participation required individuals to be: (1) 18 to 25 years of age; (2) currently enrolled as a full-time undergraduate student; (3) proficient in completing self-report measures in either Chinese or English; and (4) having electronically provided informed consent. A total of 1,570 survey responses were initially collected. To ensure data integrity, the following pre-established exclusion criteria were implemented: (1) unreasonably short completion times (< 5 min); (2) straight-line or patterned responding; (3) logical inconsistencies (e.g., simultaneous endorsement of “never exercising” and “high-intensity exercise > 3 h daily”); and (4) duplicate submissions (same IP address, student ID, or phone number), retaining only the first valid entry. After screening, 134 cases were removed, yielding 1,436 valid responses (effective completion rate = 91.5%). The final analytic sample comprised 725 males (50.50%) and 711 females (49.50%) participants. Class year distribution was as follows: 503 first-year (35.00%), 427 s-year (29.70%), 298 third-year (20.80%), and 208 fourth-year students (14.50%). The age of participants ranged from 18 to 22 years (M = 19.39, SD = 1.12). The research protocol received approval from the Institutional Review Board at Jiangxi Normal University (IRB-JXNU-PEC-2025019) and was conducted in compliance with the ethical standards outlined in the Declaration of Helsinki.

### 3.2 Measures

#### 3.2.1 Generalized Anxiety Disorder Scale (GAD-7)

Anxiety levels in the university student cohort were measured using the Generalized Anxiety Disorder 7-item scale (Spitzer et al., 2006). The tool contains seven items reflecting core anxiety symptoms, including persistent tension and uncontrollable worry. Items were rated on a 4-point frequency continuum from 0 (never) to 3 (almost daily). Composite scores were interpreted using established clinical bands: minimal (0–4), mild (5–9), moderate (10–14), and severe (15–21), wherein elevated totals correspond to more pronounced symptomology. Previous validation work supports the instrument’s reliability and validity in tertiary education contexts (Han et al., 2023). In this investigation, the GAD-7 also exhibited high scale reliability (*α* = 0.884).

#### 3.2.2 Psychological resilience scale

To measure psychological resilience in the participating collegiate cohort, the abbreviated 10-item Connor-Davidson Resilience Scale (CD-RISC-10) (Campbell-Sills and Stein, 2007) was administered. This scale consists of ten items (e.g., “Able to adapt to change” or “Tend to bounce back after illness or hardship”). Items were rated on a 5-point Likert-type scale from 0 (not true at all) to 4 (true nearly all of the time). Scores on the scale range from 0 to 40, where elevated scores are indicative of stronger psychological resilience. Previous validation supports its applicability in academic settings (Dalmış et al., 2025). In this investigation, the CD-RISC-10 also exhibited high scale reliability (*α* = 0.879).

#### 3.2.3 Physical Activity Rating Scale (PARS-3)

Physical activity among university participants was evaluated using the Physical Activity Rating Scale (Liang, 1994). This scale comprises three items (e.g., “When engaging in physical activity at the intensity as mentioned above, how many minutes do you typically spend per session?”). This instrument examines activity volume across three domains: intensity, duration, and frequency. A composite scoring algorithm was applied, incorporating ordinal response options: duration items used a 4-point scale, while intensity and frequency were rated on 5-point scales. Cumulative scores were stratified into three categories: low (0–19), moderate (20–42), and high (≥43) activity levels, with higher totals indicating greater overall activity. Previous validation supports its applicability in academic settings (Yuan and You, 2022). In this investigation, the PARS-3 also exhibited high scale reliability (α = 0.843).

### 3.3 Data analysis

Statistical analyses were conducted using SPSS 26.0 and Mplus 8.3. First, common method bias was assessed using Harman’s single-factor test. Pearson correlation analysis was employed to examine the relationships among anxiety, psychological resilience, and physical activity in university students. Next, a mediation structural equation model was constructed based on Model 4 of the PROCESS macro (Tu et al., 2021). The bootstrap method (95% confidence interval; 5,000 resamples) was applied within a linear regression framework to test whether the mediating effect of psychological resilience between anxiety and physical activity was statistically significant. For the structural equation models, the following thresholds for good model fit were applied: CFI and TLI > 0.90, RMSEA and SRMR < 0.08. Finally, LCA was performed in Mplus 8.3 to identify heterogeneous subgroups based on profiles of anxiety and psychological resilience among the students. Model fit in LCA was evaluated using the following criteria: low values of Akaike Information Criterion(AIC), Bayesian Information Criterion(BIC), and sample-size-adjusted BIC (aBIC) indicated better fit; entropy closer to 1 suggested clearer class separation; and significant results (*p* < 0.05) in both the Lo–Mendell–Rubin adjusted likelihood ratio test (LMR-LRT) and the bootstrap likelihood ratio test (BLRT) indicated that the k-class model fit significantly better than the (k-1)-class model. A significance level of *α* = 0.05 was used for all inferential tests.

## 4 Results

### 4.1 Testing for common method bias

To address potential common method variance arising from the self-report nature of the measures, Harman’s single-factor test was performed in accordance with established methodology (Podsakoff et al., 2003). An exploratory factor analysis including all items related to anxiety, psychological resilience, and physical activity revealed three factors with eigenvalues above 1.0. The most substantial factor explained 23.856% of the total variance (<40%), suggesting that common method bias does not pose a significant threat to the interpretation of the results in this study. Suggesting minimal common method bias concern in this investigation.

### 4.2 Analysis of demographic differences

Analyses of anxiety, psychological resilience, and physical activity among college students revealed pronounced gender differences; Female participants exhibited markedly elevated anxiety symptomatology relative to their male counterparts. At the same time, males exhibited greater psychological resilience and higher engagement in physical activity. In contrast, no statistically significant variations were observed across academic year groups in any of these variables (Table 1).

**Table 1:** Variations by gender and academic year in university students.

Variables	Groups	Anxiety	Psychological resilience	Physical activity
Gender	Male (725, 50.05%)	0.78 ± 0.58	2.26 ± 0.92	3.70 ± 1.10
	Female (711, 49.50%)	1.03 ± 0.78	1.96 ± 0.97	2.64 ± 1.6
	*t*	−6.72	5.90	17.82
	*p*	000^***^	000^***^	000^***^
Grade	Freshmen year (503, 35.00%)	0.88 ± 0.72	2.07 ± 0.96	3.20 ± 1.25
	Sophomores (427, 29.70%)	0.90 ± 0.66	2.15 ± 0.9	3.21 ± 1.25
	Juniors (298, 20.80%)	0.95 ± 0.67	2.08 ± 0.89	3.13 ± 1.20
	Seniors (208, 14.50%)	0.91 ± 0.78	2.20 ± 1.02	3.09 ± 1.24
	*F*	0.83	1.36	0.64
	*p*	0.46	0.25	0.59

^***^p < 0.001.

### 4.3 Correlation analysis

Anxiety demonstrated a statistically significant negative correlation with psychological resilience, and a statistically significant inverse relationship was observed with levels of physical activity. Additionally, psychological resilience was significantly positively correlated with physical activity (Table 2).

**Table 2:** Correlation analysis between variables (*N* = 1,436).

Variables	M	SD	Anxiety	Psychological resilience	Physical activity
Anxiety	0.902	0.699	1		
Psychological resilience	2.111	0.953	−0.427^***^	1	
Physical activity	3.172	1.244	−0.372^***^	0.333^***^	1

^***^p < 0.001. M: mean. SD: standard deviation.

### 4.4 The mediation effect of psychological resilience

Multicollinearity among predictors was examined by specifying physical activity as the outcome variable and anxiety and psychological resilience as predictors. All variance inflation factors remained low (Max VIF = 1.224), well under the standard cutoff of 3, indicating the absence of notable multicollinearity and supporting the robustness of parameter estimates. The model demonstrated excellent fit across multiple indices: χ^2^/DF = 1.471, RMSEA = 0.018, CFI = 0.993, NFI = 0.979, RFI = 0.976, TLI = 0.992, and GFI = 0.983. Each of these values met or exceeded accepted benchmarks for good fit, supporting its use in subsequent mediation tests.

The intermediary function of psychological resilience was evaluated employing Hayes’ PROCESS macro (Model 4) based on 5,000 bias-adjusted bootstrap iterations (95% CI). The analysis treated anxiety as the predictor, psychological resilience as the mediating variable, and physical activity as the outcome, while incorporating gender as a covariate. Findings revealed a significant negative total effect of anxiety on physical activity (*β* = −0.307, *p* < 0.001). After accounting for the mediator, the direct impact of anxiety remained statistically significant (*β* = −0.234, *p* < 0.001). Furthermore, anxiety was found to be an important negative predictor of psychological resilience (*β* = −0.413, *p* < 0.001), whereas psychological resilience exhibited a significant positive association with physical activity (*β* = 0.178, *p* < 0.001) (Table 3).

**Table 3:** Regression analysis between variables.

Variables	Model1		Model2		Model3	
	Physical activity		Psychological resilience		Physical activity	
	*β*	*t*	*β*	*t*	*β*	*t*
Anxiety	−0.307	−13.434^***^	−0.413	−17.101^***^	−0.234	−9.475^***^
Psychological resilience					0.178	7.256^***^
Gender	−0.744	0.046	−0.164	0.048	−0.715	0.045
*R*	0.522		0.435		0.546	
*R^2^*	0.272		0.189		0.299	
*F*	268.782^***^		167.143^***^		203.194^***^	

^***^p < 0.001.

**Figure 1:**
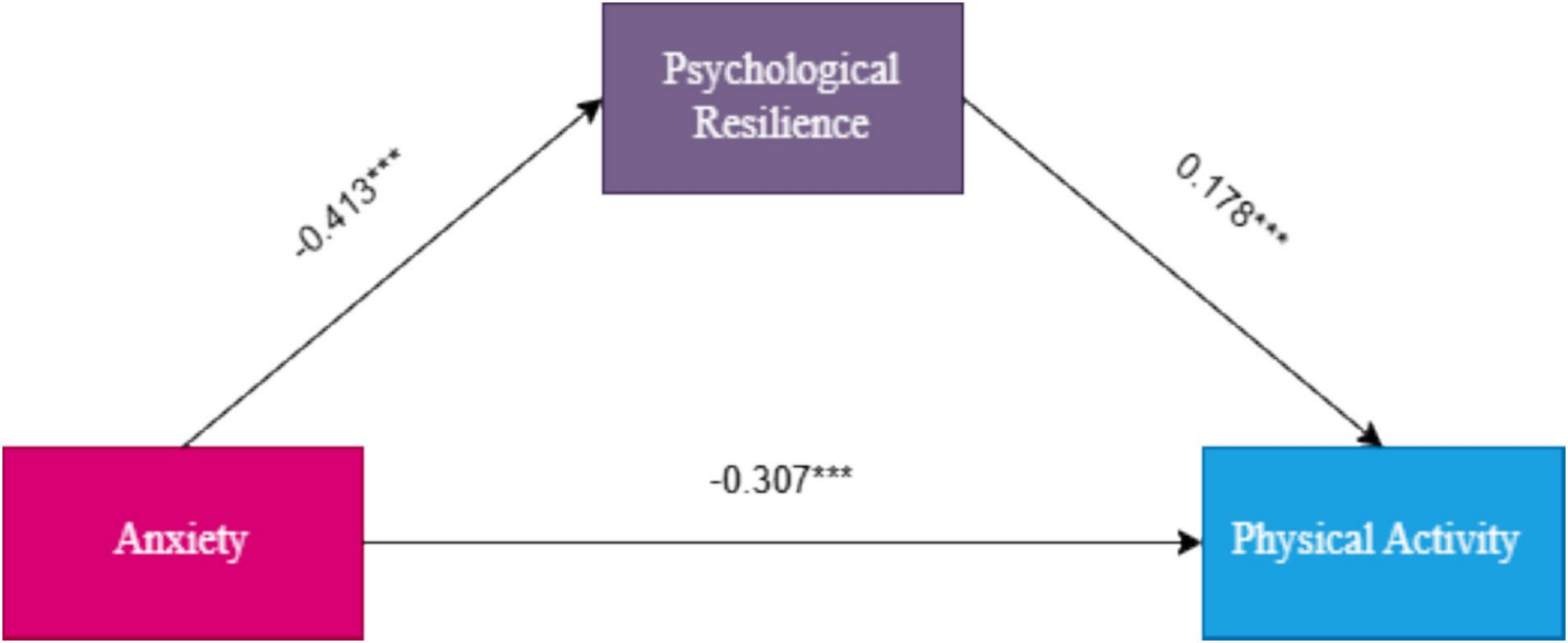
Mediation effect diagram.

The analysis further indicated a significant mediatory role of psychological resilience in the relationship between anxiety and physical activity, with an indirect effect estimate of −0.074 (95% CI: −0.099 to −0.051). This mediation accounted for 24.0% of the overall effect (Table 4). The substantial mediatory role emphasizes psychological resilience as an influential mechanism underlying the association between anxiety and physical activity among university students. These findings provide empirical support for the hypothesized mediating effect, thus confirming Hypothesis 1 (Figure 1).

**Table 4:** Mediation analysis results.

	Effect	BOOT SE	95%CI		Relativistic effect
			LLCI	ULCI	
Total effect	−0.307	0.023	−0.3533	−0.263	
Anxiety → Physical activity	−0.234	0.025	−0.282	−0.186	76%
Anxiety → psychological resilience → Physical activity	−0.074	0.012	−0.099	−0.051	24%

All coefficients are standardized. The mediation effect was tested using bias-corrected non-parametric percentile bootstrap with 5,000 resamples; 95% CI is reported. An effect is significant if the CI does not contain zero.

### 4.5 Latent profile analyses

LPA was employed to identify homogeneous subgroups characterized by differing levels of anxiety and psychological resilience among university students. The analysis incorporated responses from 1,436 participants on the GAD-7 and CD-RISC-10 instruments. Competing models with one through five latent classes were evaluated (Table 5). All information criteria (AIC, BIC, and aBIC) exhibited a progressive reduction from the one-class to the two-class model configuration. From three classes onward, the rate of improvement in these fit indices showed diminishing returns. The three-class model achieved the highest entropy value (0.939), reflecting superior classification accuracy and model interpretability compared to alternative solutions. Consequently, the class 3 model was retained as the final and most parsimonious representation of the latent subgroups.

**Table 5:** Model fit indices for the LPA of anxiety and psychological resilience among university students (*N* = 1,436).

Model	LL	AIC	BIC	aBIC	LMR (*P*)	BLRT (*P*)	Entropy	Categorical probability%
Class1	−38293.674	76655.348	76834.515	76726.508				
Class2	−36044.028	72192.055	72466.075	72300.889	<0.001	<0.001	0.887	33.43%/66.57%
Class3	−34415.365	68970.729	69339.602	69117.236	<0.001	<0.001	0.939	10.38%/62.74%/26.88%
Class4	−33782.364	67740.727	68204.454	67924.907	<0.001	<0.001	0.906	15.11%/10.52%/47.91%/26.46%
Class5	−33488.695	67189.390	67747.969	67411.243	<0.001	<0.001	0.929	13.58%/48.61%/9.89%/26.39%/1.53%

**Figure 2:**
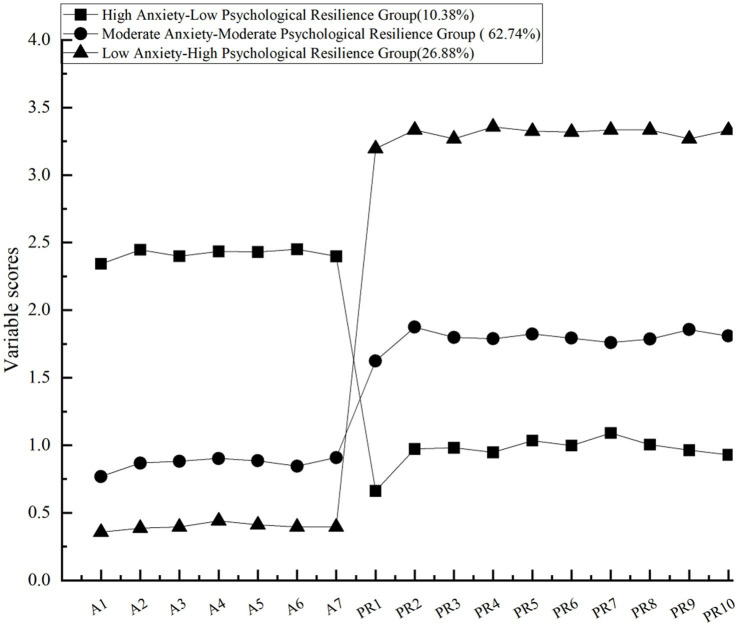
Latent classes of anxiety and psychological resilience. A1–A7 represent the 7 items for anxiety, PR1-PR10 represent the 10 items for psychological resilience.

LPA yielded three interpretable subpopulations of students, each demonstrating characteristic patterns of anxiety and resilience. Figure 2 illustrates pronounced disparities across every item of the anxiety and resilience assessment instruments among these empirically derived profiles. Based on their distinctive response patterns, the three profiles were labeled as follows: High Anxiety-Low Psychological Resilience (HA-LPR; 10.38%), Moderate Anxiety-Moderate Psychological Resilience (MA-MPR; 62.74%), and Low Anxiety-High Psychological Resilience (LA-HPR; 26.88%). These findings provide empirical support for Hypothesis 2.

### 4.6 Associations of anxiety and psychological resilience profiles with physical activity in university students

Analysis of variance revealed statistically significant differences in physical activity across the derived latent profiles based on anxiety and resilience patterns (*p*<0.001). Post-hoc comparisons revealed that all pairwise contrasts between the three groups were statistically significant. Specifically, the Low Anxiety-High Psychological Resilience Group reported the highest level of physical activity. The Moderate Anxiety-Moderate Psychological Resilience Group profile demonstrated intermediate physical activity engagement, whereas the High Anxiety-Low Psychological Resilience Group subgroup exhibited the minimal activity level among all identified groups (Table 6).

**Table 6:** Differences in physical activity across anxiety and psychological resilience profiles.

Variable	C1 (149)	C2 (901)	C3 (386)	*F*	η^2^	Back texting
Physical activity	1.494 ± 0.445	3.258 ± 1.168	3.618 ± 1.095	209.655^***^	0.226	C3 > C2 > C1

^***^p<0.001. Post hoc analyses were conducted using pairwise comparisons with a Bonferroni correction to control for Type I error across multiple comparisons. C1 represents the High Anxiety-Low Psychological Resilience group, C2 denotes the Moderate Anxiety-Moderate Psychological Resilience group, and C3 corresponds to the Low Anxiety-High Psychological Resilience group.

## 5 Discussion

This study examined the interrelationships among anxiety, psychological resilience, and physical activity in a university student sample through the use of latent profile analysis. The results indicate that psychological resilience served as a significant mediator between anxiety and physical activity. Moreover, three distinct profiles emerged based on patterns of anxiety and psychological resilience, which were further found to differ significantly in their levels of physical activity engagement.

### 5.1 The mediating role of psychological resilience

Results underscored the central mediating role of psychological resilience, which explained 24% of the total effect magnitude between anxiety and physical activity. This proportion exceeds that reported in recent meta-analyses focusing on university students in Europe and America (Qiu et al., 2025). Anxiety may impair executive functioning and reduce prefrontal inhibition of the limbic system (Page and Coutellier, 2019; Jacobs and Moghaddam, 2021; Kenwood et al., 2022). In contrast, psychological resilience may mitigate these effects by bolstering cognitive reappraisal and emotional regulation capabilities (Kay, 2016; Stover et al., 2024; Jesline et al., 2025). This mechanism aids in reestablishing equilibrium in cognitive and emotional processes, which in turn supports the conversion of intentions into actions and fosters sustained engagement in physical activity (Erickson et al., 2019; Troy et al., 2023). Among university students, anxiety not only impairs attentional control and executive functioning (Herman et al., 2020; Liu et al., 2020; Algaidi, 2025) but may also contribute to sleep disturbances (Chellappa and Aeschbach, 2022; Palagini et al., 2024), thereby undermining psychological resilience (Hou et al., 2021) and subsequently reducing engagement in physical activity (Du et al., 2025; Xu et al., 2025). Additionally, a randomized controlled trial illustrated that cognitive behavioral techniques effectively reduced anxiety associated with exercise in clinical samples, which in turn improved adherence to structured physical activity programs (Mason and Asmundson, 2023). Therefore, cultivating psychological resilience may not only function as a viable strategy for alleviating anxiety symptoms among the collegiate population but may also play an essential role in supporting their sustained engagement in regular physical activity over time.

### 5.2 Latent profile of anxiety and psychological resilience

This study employed LPA to identify subtypes of anxiety and psychological resilience among university students. After systematically comparing models with one to five classes using fit indices including AIC, BIC, aBIC, entropy, LMR-LRT, and BLRT, a class3 model was determined to be optimal, Hypothesis 2 was supported. Based on response patterns on anxiety and resilience scales, the subgroups were labeled as follows: The High Anxiety-Low Psychological Resilience Group, the Moderate Anxiety-Moderate Psychological Resilience Group, and the Low Anxiety-High Psychological Resilience Group. The HA-LPR group accounted for 10.38% of the sample and was considered high-risk, exhibiting marked anxiety symptoms, limited psychological resilience, emotional dysregulation, reduced physical activity, and delayed help-seeking behavior. These individuals require prioritized clinical intervention. The MA-MPR group, comprising 62.74% of participants, was identified as a transitional subgroup. Although not meeting criteria for acute distress, these students remain vulnerable to symptom escalation and would benefit from preventive interventions aimed at enhancing resilience. The LA-HPR group (26.88%) demonstrated strong emotional stability and adaptive capacity, representing a low-risk reference group whose behavioral and psychological profiles may inform health promotion initiatives. For the High Anxiety-Low Psychological Resilience (HA-LPR) profile, intensive individual cognitive behavioral therapy or clinical counseling is recommended to target core anxious symptomatology and systematically reconstruct coping repertoires (Zamiri-Miandoab et al., 2022; Bhattacharya et al., 2023). The Moderate Anxiety - Moderate Psychological Resilience (MA-MPR) profile would benefit from campus-wide mindfulness-based programs, stress-management workshops, and inclusive group fitness interventions designed to augment psychological resilience and forestall further deterioration (Kaplan et al., 2017; Zhang et al., 2025). Finally, individuals in the Low Anxiety-High Psychological Resilience (LA-HPR) profile should be engaged through health-promotion campaigns that consolidate existing adaptive behaviors and leverage their strengths by inviting them to serve as peer mentors (Min et al., 2012). This study provides an empirical basis for precise classification and tailored intervention strategies in university mental health promotion. In summary, the use of LPA enables a person-centred classification that moves beyond aggregate mean scores, offering a refined framework for precise intervention planning and resource allocation based on distinct anxiety and psychological resilience profiles (Lawler et al., 2020; Cao et al., 2023; Worsley et al., 2022).

### 5.3 The impact of latent profiles of anxiety and psychological resilience on university students’ physical activity

A significant association was confirmed between the identified anxiety and psychological resilience latent classes and physical activity, providing robust support for Hypothesis 3. Post-hoc comparisons revealed a pronounced stepwise gradient. The High Anxiety-Low Psychological Resilience Group (HA-LPR; 10.38%) exhibited the lowest physical activity engagement, followed by the Moderate Anxiety-Moderate Psychological Resilience Group (MA-MPR; 62.74%), whereas the Low Anxiety-High Psychological Resilience Group (LA-HPR; 26.88%) displayed the highest activity levels. These findings underscore that the anxiety severity and psychological resilience magnitude constitute a pivotal determinant of physical activity among university students. The observed empirical gradient is supported by the theoretical mechanism outlined in the Conservation of Resources Theory (Hobfoll, 1989). When individuals perceive chronic depletion of anxiety and related resources coupled with insufficient resilience reserves, their primary objective shifts from “pursuing positive health behaviors” to “preventing further loss,” manifesting as reduced engagement in physical activity. For the HA-LPR profile, low psychological resilience not only undermines self-efficacy (Bandura, 1997) but also diminishes the perceived feasibility of exercise benefits, ultimately rendering activity avoidance a seemingly resource-conserving coping strategy (Ma and Martin Ginis, 2018). Notably, the MA-MPR profile, comprising half the sample, remains below clinical anxiety thresholds, yet their “moderate anxiety and moderate psychological resilience” configuration indicates a precarious equilibrium. When confronted with intensified academic or interpersonal stressors, these individuals could rapidly transition toward the HA-LPR trajectory. Interventions for MA-MPR should therefore prioritize early resource replenishment and skill-building (e.g., brief high-intensity interval training sessions or peer-accompanied running schemes on campus) to expand psychological resilience capacity at minimal cost. Conversely, the LA-HPR group leverages high psychological resilience to buffer transient anxiety and sustain regular exercise. Beyond serving as a healthy reference, these students can be empowered as “peer mentors,” disseminating adaptive behavioral norms through social networks by sharing exercise experiences and emotion-regulation techniques (Pressman et al., 2020; Liu et al., 2024; Qureshi et al., 2024).

## 6 Limitations and future directions

Several constraints qualify the inferences that can be drawn from the current work. First, the cross-sectional nature of the data precludes any conclusive statements about temporal ordering or causation. Although mediation and latent profile modeling can generate plausible mechanistic accounts, they remain fundamentally descriptive when applied to concurrent observations. Consequently, we cannot rule out the possibility that the observed associations reflect reverse causality (e.g., individuals with higher physical activity may subsequently develop greater psychological resilience, which in turn attenuates anxiety) or the operation of unobserved reciprocal effects. Longitudinal cohort designs with repeated measurement occasions, or short-term experimental manipulations of physical activity followed by assessments of both resilience resources and anxiety symptomatology, are required to disentangle these dynamic processes.

Second, the use of self-report measures is a notable limitation, pointing to the value of integrating objective data in future studies. Future investigations should therefore triangulate subjective reports with clinician-administered diagnostic interviews. e.g., the Structured Clinical Interview for DSM-5 Disorders (SCID-5), ecological momentary assessment of affective states, and objective indicators of movement (e.g., wrist-worn accelerometry or combined heart-rate/movement sensors). Such multimethod approaches would not only attenuate common-method bias but also enable finer-grained tests of the intensity, duration, and contextual moderators that may underlie resilience-building effects of physical activity. Furthermore, other important confounding variables, such as depression, sleep quality, and social support, may also influence physical activity outcomes and should be accounted for.

Third, the cultural and demographic specificity of the sample limits external validity. All participants were full-time undergraduates recruited from universities in mainland China, a population that is relatively homogeneous with respect to age, educational attainment, and sociocultural norms. Whether the latent anxiety and psychological resilience profiles identified here generalize to community adults, clinical samples, or individuals from individualistic cultural contexts remains an open empirical question. Cross-cultural replication is particularly important given evidence that collectivistic societies may foster different coping repertoires and body-related self-schemas, which could alter the strength or even the direction of the observed associations.

## 7 Conclusion

This investigation substantiates the intermediary function of psychological resilience in the relationship between anxiety and physical activity within the university student population. Latent profile analysis identified three distinct typologies: High Anxiety-Low Psychological Resilience (HA-LPR; 10.38%), Moderate Anxiety-Moderate Psychological Resilience (MA-MPR; 62.74%), and Low Anxiety-High Psychological Resilience (LA-HPR; 26.88%), revealing a dose–response gradient in physical activity engagement.

This study elucidates the mediating role of psychological resilience in the relationship between anxiety and physical activity. The identification of three distinct student profiles provides a foundation for developing targeted interventions. For the high-risk High Anxiety-Low Psychological Resilience (HA-LPR) profile, we recommend implementing specialized cognitive-behavioral interventions that simultaneously address core anxiety symptoms while reconstructing adaptive coping capabilities. Furthermore, the high-functioning Low Anxiety-High Psychological Resilience (LA-HPR) group can be leveraged as peer facilitators to co-design physical activity initiatives and disseminate success narratives through institutional channels, thereby amplifying positive normative influence. Additionally, these findings offer valuable insights for university health promotion systems. Higher education institutions can develop adaptive recommendation algorithms based on the characteristics of these three profiles to enable targeted delivery of intervention content through campus health platforms, thus advancing the precision and systematization of university health services.

## Data Availability

The original contributions presented in the study are included in the article/supplementary material, further inquiries can be directed to the corresponding authors.
